# Supplementary material for: Spin Glass State and Griffiths Phase in van der Waals Ferromagnetic Material Fe5GeTe2
Source: Nanomaterials (Basel). 2024 Dec 27;15(1):19. doi: 10.3390/nano15010019 (PMC11723351; doi:10.3390/nano15010019)
Supplement: Supplementary file 1 [file nanomaterials-15-00019-s001.zip › nanomaterials-3338649-supplementary.pdf]

Supplemental Material for

Spin Glass State and Griffiths Phase in van der Waals Ferromagnetic Material Fe_5GeTe_2

Jiaqi He, Yuan Cao, Yu Zou, Mengyuan Liu, Jia Wang, Wenliang Zhu and Minghu Pan *

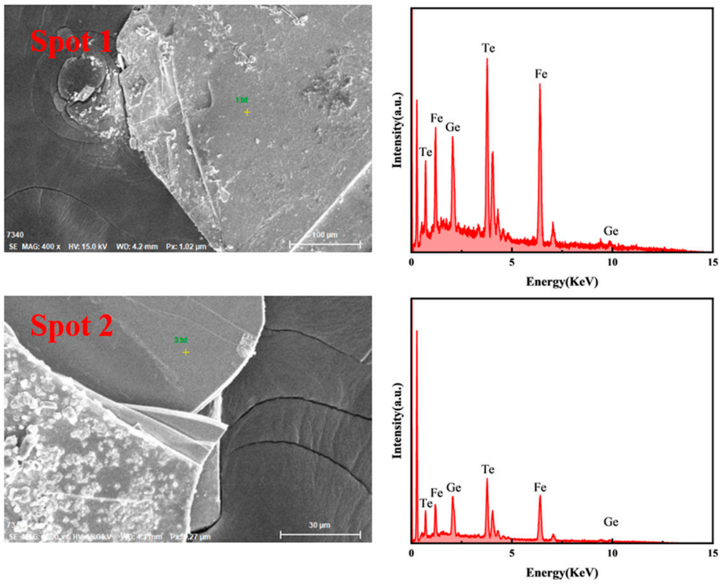

Figure S1. Several typical EDX measurement results of the Fe_5GeTe_2 single crystal.

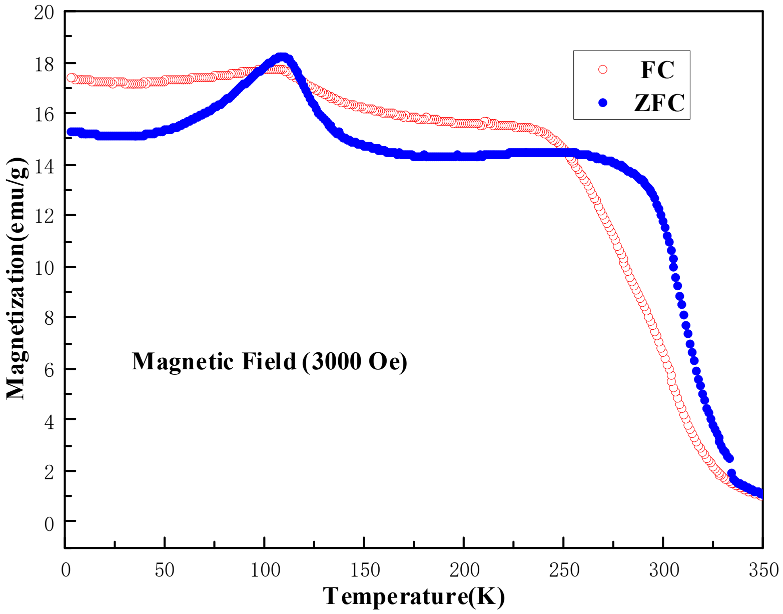

Figure S2. The plot of raw data of M-T curves of Fe_5GeTe_2 single crystal at 3000 Oe magnetic field.

Table S1. Comparison of experimental data and theoretical calculations of XRD for Fe_5GeTe_2 single crystals.

XRD peak	Generated from cif file	experiment
(003)	9.064	8.9991
(006)	18.185	18.1151
(009)	27.424	27.3061
(0012)	36.849	36.8201
(0015)	46.54	46.5381
(0018)	56.599	56.6001
(0021)	67.159	67.1781

Table S2. The averaged values of the EDX results of the Fe_5GeTe_2 single crystal.

Element number	Element Symbol	Element Name	Atomic Cons.	Weight Cons.
26	Fe	Iron	60.22	43.15
52	Ge	Tellurium	11.71	10.91
32	Te	Germanium	28.07	45.94
